# Imaging Findings of Concomitant Pulmonary and Central Nervous System Sarcoidosis: A Case Report

**DOI:** 10.4314/ejhs.v32i3.23

**Published:** 2022-05

**Authors:** Tewodros Endale Balcha, Abebe Mekonnen Woldeyohannes, Azmera Gissila Aboye

**Affiliations:** 1 Department of Radiology, College of Health Sciences, Addis Ababa University, Addis Ababa, Ethiopia

**Keywords:** Sarcoidosis, Neurosarcoidosis, Magnetic resonance imaging

## Abstract

**Background:**

Sarcoidosis is a multisystem idiopathic granulomatous disorder characterized by the development of noncaseating infiltrative granulomas in various body organs. The central nervous system (CNS) is one of the sites to be affected by sarcoidosis. We present a case of sarcoidosis with concomitant involvement of lung and CNS with emphasis on neuroimaging findings.

**Case:**

A 45-year-old Ethiopian male patient was sent to our radiology department at Tikur Anbessa Specialized Hospital for chest computed tomography (CT) and brain Magnetic resonance imaging (MRI) evaluation for an indication of panhypopituitarism, nasal congestion, and decreased vision. The chest CT reveals the perilymphatic distribution of multiple soft tissue attenuating pulmonary nodules which is a pattern seen in sarcoidosis. The brain MRI also revealed thickening and gadolinium enhancement of hypothalamus/optic chiasm which is also a common site of involvement in neurosarcoidosis. The nasal biopsy finding of granulomatous inflammation with the above imaging findings supports the diagnosis of sarcoidosis.

**Conclusion:**

As sarcoidosis is a systemic disease that can affect any organ in the body, multimodality imaging is important in the diagnosis of sarcoidosis. Brain MRI with gadolinium contrast is a preferred imaging modality that can assess different patterns and areas of CNS involvement in sarcoidosis.

## Introduction

Sarcoidosis is a multisystem idiopathic granulomatous disorder characterized by the development of noncaseating infiltrative granulomas in various body organs. It is a global disease with a prevalence of 0.2–64 cases per 100,000 people (1).

The clinical presentation of sarcoidosis is variable ranging from asymptomatic to nonspecific symptoms such as generalized fatigue, nonspecific respiratory symptoms such as cough, dyspnea, chest pain, ocular abnormalities, cutaneous lesions, peripheral lymphadenopathy, joint symptoms, etc (1,2). “The diagnosis of sarcoidosis is not standardized, but based on three major criteria: a compatible clinical presentation, finding nonnecrotizing granulomatous inflammation in one or more tissue samples, and the exclusion of alternative causes of granulomatous disease” as detailed and suggested by ATS (American Thoracic Society) guidelines for sarcoidosis diagnosis (3).

Imaging is an effective method of diagnosis, such as chest X-ray, high-resolution computer tomography (HRCT), magnetic resonance with gadolinium contrast (MRI), and/or positron emission tomography with fluorodeoxyglucose(FDG-PET).

Extrapulmonary sarcoidosis may coexist with pulmonary sarcoidosis, can overtake it, or occur following remission of the pulmonary form, sometimes after several years (4).

Neurosarcoidosis affects less than 10% of all patients with sarcoidosis and may involve any part of the nervous system. Isolated neurological involvement is rare and maybe as low as 1%. The most frequently affected sites are the cranial nerves (55%), the meninges (12–40%), the brain parenchyma (20–45%), and the spinal cord (18–26.5%). When involved, the majority (up to 75%) initially present with neurological symptoms (2, 5). The clinical features of neurosarcoidosis depend on the site of involvement and include facial nerve palsy, vision loss, diplopia, headache, seizure, meningism, weakness, and paresthesia (1). We report a case of imaging findings of a patient with the concomitant central nervous system (CNS) and pulmonary sarcoidosis, which is to our knowledge, the first image-based case reported from Ethiopia.

## Case Presentation

A 45-year-old Ethiopian male patient was sent to our radiology department at Tikur Anbessa Specialized Hospital for chest CT and brain MRI imaging evaluation for an indication of panhypopituitarism, nasal congestion, and vision difficulties.

**Figure 1:**
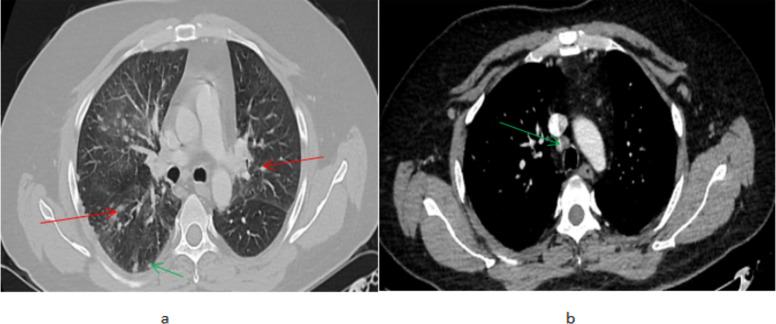
High-resolution axial chest CT (a) lung window showing bilateral different sized soft tissue attenuating pulmonary parenchymal nodules (red arrows) and a subpleural nodule on the right side (green arrow) (b) mediastinal window showing mildly enlarged right paratracheal mediastinal lymph node (green arrow)

The chest CT findings include multiple bilateral upper and lower lobes different sized soft tissue attenuating subpleural and parenchymal nodules. There is also bilateral nodular fissural thickening with a beaded appearance (Figure 1). The findings are distributed in a perilymphatic pattern which is seen in pulmonary sarcoidosis. There are also multiple subcentimeter rounded lymph nodes involving the prevascular, aortopulmonary, lower paratracheal, and bilateral hilar stations.

**Figure 2:**
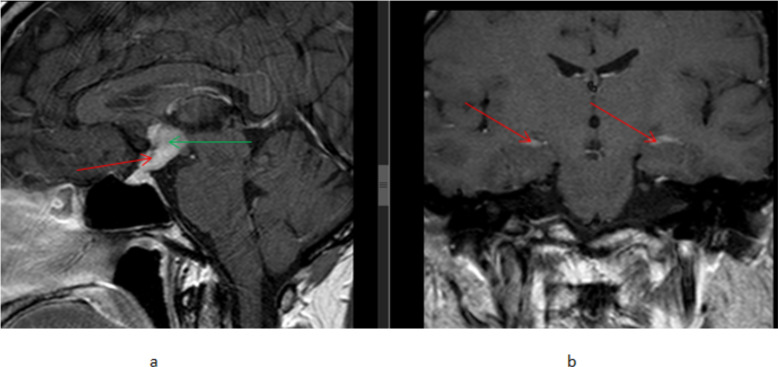
T1 brain MRI with gadolinium contrast, (a) sagittal plane showing marked mass-like thickening and diffuse gadolinium enhancement of the pituitary stalk (red arrow) and optic chiasm/hypothalamus (green arrow) (b) coronal view showing diffuse bilateral leptomeningeal thickening and gadolinium enhancement in bilateral medial temporal lobes (red arrows).

The brain MRI revealed 2.5cm*2.2cm*1.8cm measuring suprasellar nodular lesion involving the hypothalamus and pituitary stalk, prechiasmatic part of optic nerves, and optic chiasm (Figure 2). The mass shows T1 iso &T2 heterogeneously hypointense signal with avid homogeneous gadolinium contrast enhancement. There is also adjacent meningeal thickening and gadolinium enhancement. The normal gadolinium-enhancing pituitary gland was visualized.

The patient underwent endoscopic nasal evaluation and biopsy which revealed non-necrotizing granulomatous inflammation.

## Discussion

The clinical presentation of our patient which is panhypopituitarism, nasal congestion, and vision difficulties with nasal biopsy histologic findings of granulomas inflammation suggests sarcoidosis. The chest CT findings of perilymphatic pulmonary nodules and brain MRI findings of the diffuse pituitary stalk and hypothalamus/optic chiasm mass-like thickening and gadolinium enhancement with the presence of leptomeningeal thickening in the absence of known malignancy in the patient's workup further supports the diagnosis of sarcoidosis with concomitant pulmonary and CNS involvement.

Similar to our case, hypothalamus/optic chiasm and meningeal involvement are said to be among common CNS sites in neurosarcoidosis(1,4). Cranial nerve involvement is common in neurosarcoidosis, especially facial and optic nerve involvement, in which facial palsy is the most common cranial nerve palsy clinically, but on imaging optic nerve involvement is more common, like in our case (6). Isolated central nervous system sarcoidosis is rare, affecting less than 1% of all patients with sarcoidosis (1). In support of this, our patient has extra neural imaging findings which are pulmonary and mediastinal involvement.

Our case reveals imaging findings of meningeal, hypothalamus/optic nerve, and pulmonary involvement of sarcoidosis. This in turn points out the important role of multimodality imaging in the diagnosis of sarcoidosis.
